# Mechanisms of In Situ Growth of (Fe_4_Al_3_Cr)_0.25_TiO_5_ Whisker Driven by Oxygen Partial Pressure and Reinforced Mechanical Properties in Corundum‐Type Medium‐Entropy Oxides

**DOI:** 10.1002/advs.202508378

**Published:** 2025-07-30

**Authors:** Wenxue Wang, Kang Wang, Chao Ma, Wei Yang, Junpeng Jiang, Rui Zhao, Daoyang Han, Hailong Wang, Rui Zhang

**Affiliations:** ^1^ School of Materials Science and Engineering Zhengzhou University Zhengzhou Henan 450001 China; ^2^ Zhongyuan Critical Metal Laboratory Zhengzhou University Zhengzhou Henan 450001 China; ^3^ State Key Laboratory of Structural Analysis Optimization and CAE Software for Industrial Equipment Zhengzhou University Zhengzhou Henan 450002 China; ^4^ Zhengzhou Research Institute for Abrasives & Grinding Co., Ltd. Zhengzhou Henan 450001 China; ^5^ State Key Laboratory of Inorganic Synthesis and Preparative Chemistry College of Chemistry Jilin University Changchun 130012 China

**Keywords:** corundum‐type medium‐entropy oxide, first‐principles calculations, oxygen partial pressure, whisker reinforcement

## Abstract

Designing in situ whiskers to enhance the fracture toughness of ceramics presents challenges for structural applications. Herein, two corundum‐type medium‐entropy oxide ceramics (MEO‐1 and MEO‐2) are synthesized through solid‐state reaction sintering under controlled oxygen partial pressures. MEO‐1 is a single‐phase corundum‐type oxide (Al_0.41_Cr_0.26_Fe_0.31_Ti_0.02_)_2_O_3_, while MEO‐2 incorporates (Fe_4_Al_3_Cr)_0.25_TiO_5_ whiskers to reinforce (Al_0.40_Cr_0.25_Fe_0.30_Ti_0.05_)_2_O_3_. By regulating variable valence ions at different oxygen partial pressures, the whiskers achieved a maximum average length of 28.3 µm and a length‐to‐diameter ratio of 17.7 at 50.6 kPa, significantly enhancing the fracture toughness of the MEO‐2, which possesses the optimal flexural strength, Vickers hardness, and fracture toughness of 321 ± 8 MPa, 22.4 ± 1.5 GPa, and 3.87 ± 0.12 MPa m^1/2^, respectively. Bravais–Friedel–Donnay–Harker law (BFDH) simulation elucidates that oxygen partial pressures can effectively regulate ionic diffusion behavior and whisker growth. First‐principles calculations demonstrate that the whisker growth direction [0 4 0] aligns with the high shear modulus direction, contributing to the strengthening mechanism. This work provides new insights for designing high‐performance medium/high‐entropy ceramics, highlighting the critical role of oxygen partial pressure in regulating whisker growth and improving mechanical properties.

## Graphical abstract

In corundum‐type medium‐entropy oxides, oxygen partial pressure regulation facilitated the in situ growth of whiskers, significantly enhancing mechanical properties. The growth mechanism and reinforcing effects of whiskers are systematically elucidated through a combination of theoretical calculations and experimental validation, providing a novel insight for advanced structural ceramics design and applications.

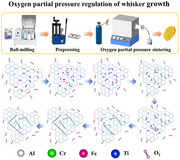

## 1 Introduction

Medium/high‐entropy ceramics (HECs), which are solid solutions of inorganic compounds with one or more Wyckoff sites shared by multi‐principal elements with equal or near‐equal atomic ratios,^[^ ^1^ ^]^ have been widely investigated in many fields such as microwave absorption,^[^ ^2^ ^]^ electromagnetic,^[^ ^3^ ^]^ energy storage,^[^ ^4^ ^]^ catalysis,^[^ ^5^ ^]^ thermal protection,^[^ ^6^ ^]^ and cutting abrasives,^[^ ^7^ ^]^ due to their distinctive high‐entropy effect, lattice distortion, hysteresis diffusion effect and “cocktail effect.”^[^ ^8^ ^]^ Unfortunately, medium/high‐entropy ceramics with corundum structure have unsatisfactory fracture toughness,^[^ ^9^ ^]^ sometimes even lower than that of the individual raw material, which confines their further application. Currently, some common strategies for improving fracture toughness are widely utilized such as secondary toughening particle phase,^[^ ^10^ ^]^ whiskers,^[^ ^11^ ^]^ and fibers toughening.^[^ ^10^, ^12^ ^]^ For instance, Luo et al. prepared SiC_w_ whisker‐toughened (Ti_0.2_Zr_0.2_Hf_0.2_Nb_0.2_Ta_0.2_)C with a fracture toughness of 4.3 ± 0.3 MPa m^1/2^, which was ≈43% higher than that of the monolithic (Ti_0.2_Zr_0.2_Hf_0.2_Nb_0.2_Ta_0.2_)C ceramic (3.0 ± 0.2 MPa m^1/2^).^[^ ^11a^ ^]^ Peter et al. fabricated (Ti_0.2_Zr_0.2_Hf_0.2_Nb_0.2_Ta_0.2_)B_2_ composited with different β‐SiC content (5, 10, 15, 20, and 25 vol%) particles and found that the maximum fracture toughness of HEB composites could reach to 4.12 ± 0.2 MPa m^1/2^.^[^ ^13^ ^]^ These methods can significantly improve the fracture toughness of medium/high‐entropy ceramics, but they are accompanied with drawbacks such as high cost, complicated preparation process, nonhomogeneous distribution, and easy segregation of additions.^[^ ^14^ ^]^

An alternative method is in situ whisker growth as a second phase, which can avoid the problems of incompatibility and uneven distribution of additions, further reducing cost and simplifying the preparation process.^[^ ^15^ ^]^ From the perspective of preparation methods, the second phase of medium/high‐entropy ceramics is mainly regulated by element composition, sintering method, and sintering temperatures.^[^ ^16^ ^]^ Oxygen partial pressure is an effective method for regulating defect concentration,^[^ ^17^ ^]^ phase composition,^[^ ^18^ ^]^ and grain growth rate in materials.^[^ ^19^ ^]^ However, its application in controlling the morphology and structure of medium/high‐entropy oxides remains underexplored. Due to the multicomponent nature and tunability of medium/high‐entropy oxides, oxygen partial pressure holds significant potential for modulating non‐equivalent ionic solid solutions and structural defects. Furthermore, the cations of oxides with corundum structure (e.g., Fe_2_O_3_ and Ti_2_O_3_),^[^ ^20^ ^]^ which possesses diverse valence states and oxygen vacancies, can be easily adjusted by oxygen partial pressure. Therefore, corundum‐type medium‐entropy oxides can be designed and synthesized by regulating oxygen partial pressures, with in situ formed whiskers acting as a second reinforcing phase, aimed at enhancing their mechanical properties.

In this study, a single‐phase corundum‐type medium‐entropy oxide (Al_0.41_Cr_0.26_Fe_0.31_Ti_0.02_)_2_O_3_ (MEO‐1) and biphasic corundum‐type medium‐entropy oxides (Al_0.40_Cr_0.25_Fe_0.30_Ti_0.05_)_2_O_3_ (MEO‐2) were prepared by solid‐phase reaction method. The influence of oxygen partial pressures on their phase compositions, microstructures, and mechanical properties was investigated. The growth mechanism of in situ formed whiskers and strengthening mechanisms were analyzed and proposed based on X‐ray photoelectron spectroscopy (XPS), electron backscattered diffraction (EBSD), high‐resolution transmission electron microscopy (HRTEM) and theoretical calculation (DFT). The in situ formed whiskers regulated by oxygen partial pressures may provide new insights for enhancing the fracture toughness of medium/high‐entropy oxide ceramics.

## 2 Experimental Section

### 2.1 Design and Synthesis of Medium‐Entropy Corundum‐Type Oxides

**Figure 1:**
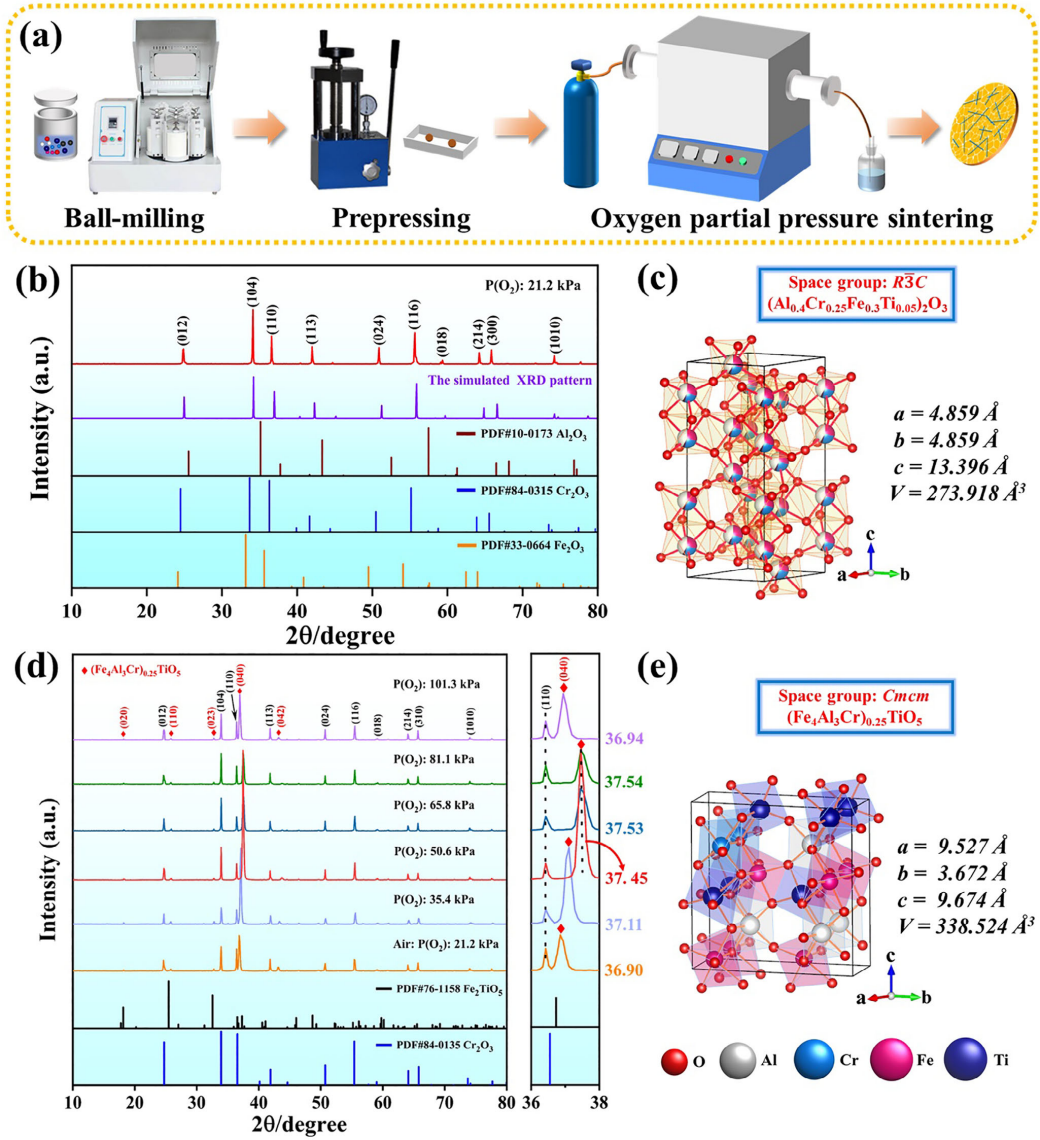
a) Schematic diagram of the samples sintering under oxygen partial pressure, b) the XRD pattern of MEO‐1, c) corundum structure (medium‐entropy phase), d) the XRD pattern of MEO‐2 prepared under different oxygen partial pressures with partial enlargement at the 2*θ* positions of 36–38°, and e) pseudo‐brookite structure (the second phase).

MEO‐1 and MEO‐2 were designed and prepared by solid‐state reaction method. The lattice parameters and cation radii were considered as main criteria during the preparation of medium‐entropy corundum‐type oxides.^[^ ^21^ ^]^ Al_2_O_3_, Cr_2_O_3_, Fe_2_O_3_, and Ti_2_O_3_ were selected as the constituting binary oxides and their lattice parameters are summarized in Table S1 (Supporting Information) based on the Material Project database (https://materialsproject.org). However, Ti_2_O_3_ was unstable at high temperatures and can easily convert to other oxides (TiO_2_, Ti_3_O_5_, Ti_4_O_7_).^[^ ^22^ ^]^ To avoid the above issues, TiO_2_ was selected to replace Ti_2_O_3_ so that stable medium‐entropy oxides can be formed. Meanwhile, the content of Ti element has a significant effect on the formation of single‐phase solid solution of corundum‐type oxides because the maximum solid solubility of TiO_2_ in alumina was only 2–4 at%.^[^ ^23^ ^]^ When the Ti content reaches 5 at% in the system, the second phase may be generated. Commercially available α‐Al_2_O_3_, α‐Fe_2_O_3_, Cr_2_O_3_, and rutile (TiO_2_) powders (99.9% purity, Rhawn Biochemical Co., Ltd., Shanghai, China) were purchased as raw materials. The raw powders weighed with targeted compositions were ball‐milled for 12 h at 300 rpm with the ball‐to‐material ratio of 4:1. After ball milling, the homogeneous slurries were dried by a rotary evaporator. The obtained mixture was screened using a 200‐mesh sieve and pressed uniaxially for 60 s under the pressure of 25 MPa. Finally, the green bodies were calcined at 1500 °C for 2 h under different oxygen partial pressures (21.2, 35.4, 50.6, 65.8, 81.1, and 101.3 kPa). The schematic diagram of the preparation process by regulating oxygen partial pressure sintering is shown in **Figure** **1**a.

### 2.2 Characterization

The phase compositions of the samples were analyzed by an X‐ray diffractometer (XRD, D8 Advanced, Bruker, Germany) with Cu Kα radiation at a step size of 0.02° and a scanning rate of 5° min^−1^. The chemical valence states and oxygen vacancy contents were analyzed by an X‐ray photoelectron spectrometer (XPS, Thermo Kalpha, USA). The microstructures of the specimens were observed by a scanning electron microscope (SEM, Zeiss Sigma 300, UK) equipped with an electron backscattered diffraction detector (EBSD, Nordly max3, UK). High‐resolution transmission electron microscopy (HRTEM, FEI Tecnai F20, USA) was employed to study the detailed microstructure of MEO‐2. The bulk densities of the samples were measured according to Archimedes drainage method. The Vickers hardness of the specimens was determined using a Vickers hardness tester (AHVD‐1000XY, Shanghai, China) under an applied load of 0.5 N and a dwelling time of 15 s. The Young's modulus and nano‐hardness of the MEO‐2 were measured using a nano‐indenter (Bruker Hysitron TI980, USA). The specimens were cut into 16 × 3 × 2 mm^3^ strips (length × width × thickness) and polished with 1.3 µm diamond grinding paper. The bending strength of the specimens was tested by the three‐point bending method (the support span was set at 10 mm and the crosshead speed was 0.5 mm min^−1^). The fracture toughness was tested via the single‐edge notched beam specimens (SENB) in three‐point bending (the support span was set at 16 mm and the crosshead speed was 0.5 mm min^−1^) and the sizes of the samples were 22 × 2 × 4 mm^3^ (length × width × thickness) with a notch depth of 2 mm and a width of 0.2 mm.

### 2.3 Theoretical Calculation on Strengthening Mechanisms

To investigate the strengthening mechanism of the second phase on the mechanical properties of MEO‐2, first‐principles calculations based on the density functional theory (DFT) were employed and accomplished by the Vienna Ab‐Initio Simulation Package (VASP‐5.4.4).^[^ ^24^ ^]^ The core‐valence electron interactions were described by the projected‐augmented wave (PAW) method and the exchange‐correlation function was processed by the generalized gradient approximation with the Perdew–Burke–Ernzerhof (GGA‐PBE).^[^ ^25^ ^]^ To simulate the disorder of the cations in the lattice, the special quasi‐random structure (SQS) approach was applied to construct the corundum supercell (the supercell contains 24 metal atoms and 36 oxygen atoms) by the Alloy Theoretic Automated Toolkit (ATAT) package.^[^ ^26^ ^]^ (Fe_4_Al_3_Cr)_0.25_TiO_5_ supercell totally contains 32 atoms including 4 Fe atoms, 3 Al atoms, 1 Cr atom, 4 Ti atoms, and 20 O atoms. During the DFT calculations, the plane‐wave cut‐off energy of 520 eV and the k‐mesh grid of 0.02 Å^−1^ based on Monkhorst–Pack scheme were used for geometry optimization and mechanical property calculation.^[^ ^27^ ^]^ The electronic energy convergence criterion and the ionic force convergence criterion were set at 10^−5^ eV and 0.02 eV Å^−1^, respectively.^[^ ^28^ ^]^ Moreover, Grimme's semiempirical DFT‐D3 scheme of dispersion correction method has been incorporated to account for the long‐range van der Waals (vdW) interactions.^[^ ^29^ ^]^ A vacuum layer was constructed as 15 Å to avoid interaction between neighboring layers.^[^ ^30^ ^]^

## 3 Results and Discussion

### 3.1 Structural Characterization

Figure 1b shows the XRD pattern of MEO‐1 prepared at an oxygen partial pressure of 21.2 kPa. All diffraction peaks are consisted with the simulated XRD pattern calculated through the Reflex code of the Materials Studio software package (Materials Studio 2019, Accelrys, USA), indicating that a single‐phase medium‐entropy solid solution was prepared successfully. However, the position of some peaks for MEO‐1 has a deviation compared to those of corundum‐type standard PDF cards, which should be caused by the different atomic scattering factors of solid solution elements (more details are explained in Figure S1, Supporting Information). The crystal structure of MEO‐1 is corundum, belonging to the trigonal crystal system with a space group of *R* $\bar{3}$ *C* (Figure 1c). Figure 1d shows the XRD patterns of MEO‐2 prepared under different oxygen partial pressures. Compared to the Cr_2_O_3_ PDF card (PDF#84‐0135), several weak peaks at 18.25°, 25.87°, 32.73°, and 43.72° should be attributed to the formed second phase, which is determined to be (Fe_4_Al_3_Cr)_0.25_TiO_5_ with orthorhombic structure (Fe_2_TiO_5_, PDF#76‐1158), as shown in Figure 1e. Additionally, we performed XRD pattern simulations of (Fe_4_Al_3_Cr)_0.25_TiO_5_ by geometric optimization and experimentally synthesized (Fe_4_Al_3_Cr)_0.25_TiO_5_. The results reveal that (Fe_4_Al_3_Cr)_0.25_TiO_5_ has an orthorhombic structure with the *Cmcm* space group. Moreover, the replacement of Fe with Al and Cr, which possess smaller ionic radii, results in a shift of the diffraction peaks toward higher angles relative to Fe_2_TiO_5_, as depicted in Figure S2 (Supporting Information). Noticeably, with the increasing oxygen partial pressure, the positions of the (0 4 0) plane are located at 36.90°, 37.11°, 37.45°, 37.53°, 37.54°, and 36.94°, respectively. The diffraction peak for the (0 4 0) plane exhibited the largest shift to a higher angle and the strongest intensity at the oxygen partial pressure of 50.6 kPa, which could be related to the preferred orientation of the second phase,^[^ ^31^ ^]^ significantly affected by oxygen partial pressures.

**Figure 2:**
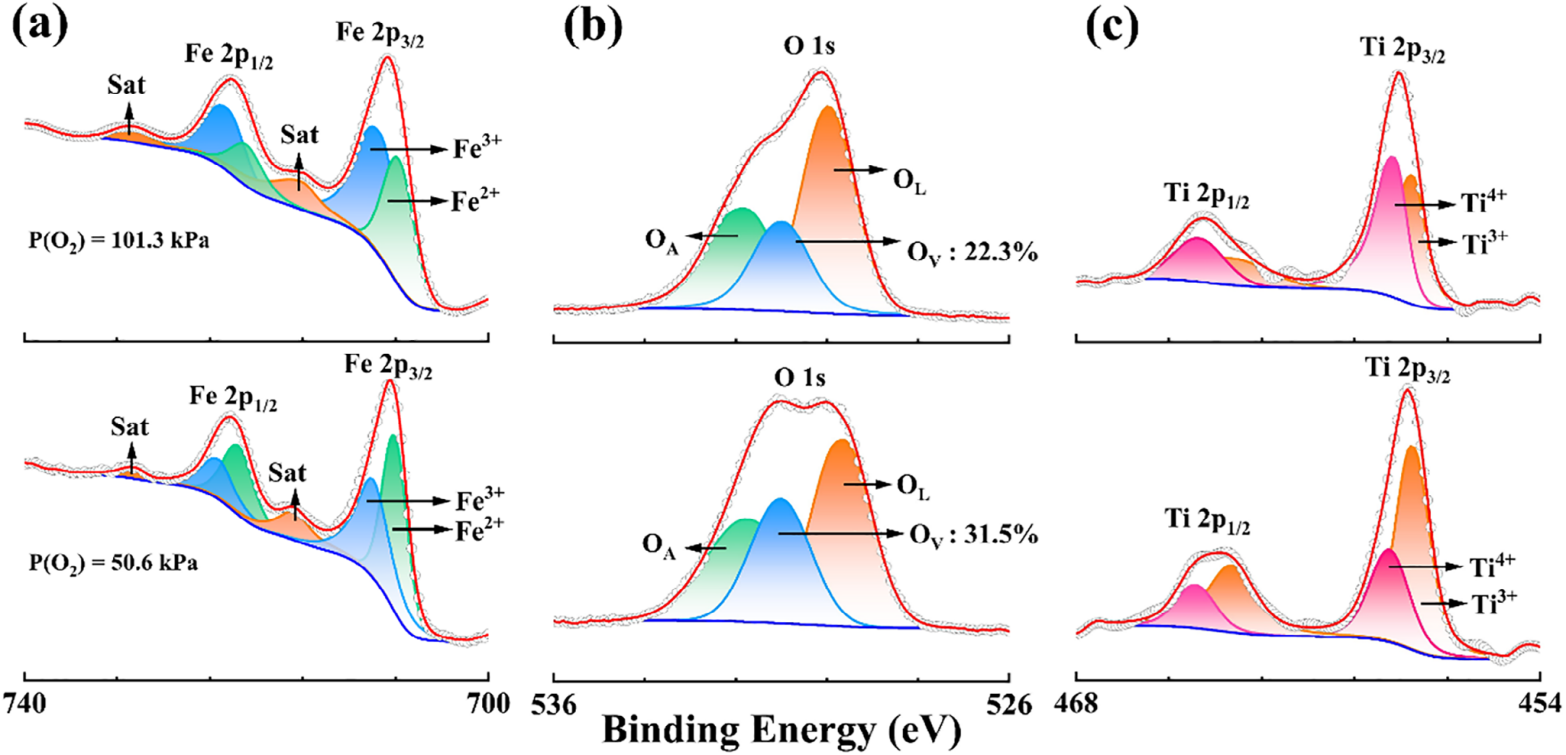
a) Fe 2p, b) O 1s, and c) Ti 2p XPS spectra of MEO‐2 prepared under oxygen partial pressures of 50.6 and 101.3 kPa.

To explore the effect of the oxygen partial pressures on the structure of MEO‐2, X‐ray photoelectron spectroscopy was employed to analyze the chemical states of the elements. Figure S3 (Supporting Information) shows the XPS full spectrum of MEO‐2 prepared at 50.6 and 101.3 kPa. It can be seen that MEO‐2 contains Al, Cr, Fe, Ti, and O elements. **Figure** **2**a shows the Fe 2p spectrum of MEO‐2, which was deconvoluted into four spin‐orbital peaks and two satellite peaks.^[^ ^32^ ^]^ The ratio of Fe^3+^ and Fe^2+^ increased when the partial pressure of oxygen increased from 50.6 to 101.3 kPa. The formed Fe^2+^ should be generated by the decomposition of Fe_2_O_3_ at insufficient oxygen partial pressures, which can be expressed by the following equations:^[^ ^33^ ^]^

(1) $$6Fe_{2}O_{3}\overset{{1350\;}^{\circ }C}{\to }4Fe_{3}O_{4}+O_{2}↑$$

(2)

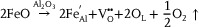

where O_L_ and $V_{O}^{••}$ represent lattice oxygen and oxygen vacancy, respectively. Based on the solid solution reaction Equation (2), it can be speculated that the increased oxygen partial pressure constricts the generation of oxygen vacancy defects. The O 1s spectrum can be deconvoluted to three peaks (Figure 2b), which are assigned to the lattice oxygen (O─M─O in metal oxide, O_L_), oxygen vacancy (V_O_), and absorbed oxygen on the sample surface (O_A_).^[^ ^34^ ^]^ The relative content of oxygen vacancies decreased from 26.3% to 19.4% when the oxygen partial pressure increased from 50.6 to 101.3 kPa, confirming that oxygen vacancies can be effectively regulated by adjusting oxygen partial pressure. Furthermore, the Ti 2p spectrum is displayed in Figure 2c. Two fitted peaks corresponding to Ti 2p_1/2_ and Ti 2p_3/2_ can be attributed to Ti^3+^ and Ti^4+^.^[^ ^35^ ^]^ The possible solid solution reactions are described by the following equations:^[^ ^23^, ^36^ ^]^

(3)

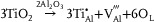

(4) $$2\mathrm{Ti}O_{2}\overset{\mathrm{Low}\;P(O_{2})}{\to }2Ti^{3+}{+V}_{O}^{••}+3O^{2-}+2e^{\prime }+\frac{1}{2}O_{2}↑$$

(5) $$Ti^{3+}+3O^{2-}\overset{Al_{2}O_{3}}{\to }{\left(Ti_{\mathrm{Al}}\right)}_{2}O_{3}$$

According to the above equations, the increasing oxygen partial pressure promotes Ti^4+^ solid solution, suppressing Ti^3+^ formation. Therefore, the oxygen partial pressure has a significant effect on the content of oxygen vacancy and chemical states of metal elements (Ti^3+^ (r = 0.670 Å), Ti^4+^ (r = 0.605 Å), Fe^2+^ (r = 0.780 Å), Fe^3+^(r = 0.645 Å)),^[^ ^37^ ^]^ which further affect the structure and morphology of (Fe_4_Al_3_Cr)_0.25_TiO_5_ phase.

**Figure 3:**
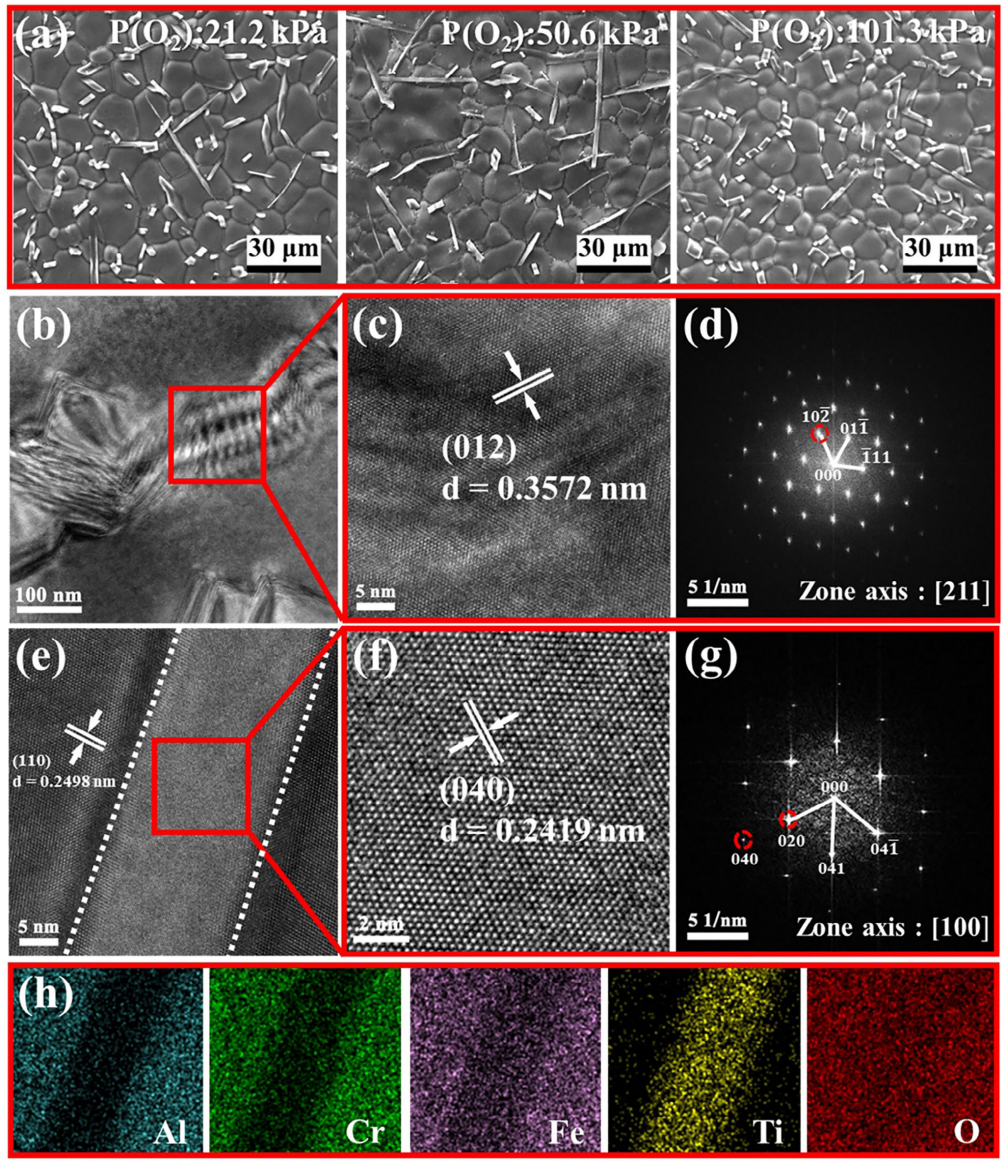
a) The surface morphologies of MEO‐2 under different oxygen partial pressures, b) TEM image and c) HRTEM image of corundum phase in MEO‐2 prepared under 50.6 kPa, d) the Fast Fourier Transform (FFT) morphology of its HRTEM image, e,f) HRTEM images of the pseudo‐brookite phase, g) the Fast Fourier Transform (FFT) morphology, and h) the EDS mapping.

**Figure** **3**a shows the surface morphologies of MEO‐2 prepared at oxygen partial pressures of 21.2, 50.6, and 101.3 kPa, respectively. It can be observed that the formed (Fe_4_Al_3_Cr)_0.25_TiO_5_ phase in MEO‐2 has whisker morphologies with inhomogeneous length, which are mainly distributed at grain boundaries. While MEO‐1 does not contain a whiskers structure (Figure S4, Supporting Information). The chemical compositions of (Al_0.40_Cr_0.25_Fe_0.30_Ti_0.05_)_2_O_3_ and (Fe_4_Al_3_Cr)_0.25_TiO_5_ are shown in Figure S5 (Supporting Information), and are essentially consistent with the chemical formula and the XRD results. Furthermore, the whiskers in MEO‐2 prepared at oxygen partial pressures of 50.6 kPa possessed a larger average length and aspect ratio (Figure S6, Supporting Information). Consequently, high‐resolution TEM (HRTEM) was applied to further confirm the microstructure of MEO‐2 prepared at an oxygen partial pressure of 50.6 kPa. Figure 3b shows the TEM image of (Al_0.40_Cr_0.25_Fe_0.30_Ti_0.05_)_2_O_3_ and ultra‐dense dislocations can be observed, which is beneficial for the improvement of strength and hardness. The measured lattice spacings of 0.3572 nm correspond well to (0 1 2) lattice plane of (Al_0.40_Cr_0.25_Fe_0.30_Ti_0.05_)_2_O_3_. Based on the SAED pattern (Figure 3d), the well‐arranged diffraction spots along the zone axes [2 1 1] indicate that MEO‐2 possesses a corundum structure. Meanwhile, Figure 3e,f displays the HRTEM images of (Fe_4_Al_3_Cr)_0.25_TiO_5_ phase, whose atoms are neatly arranged without lattice distortion. The lattice fringes with interplanar spacings of 0.2419 nm correspond to the (0 4 0) plane of (Fe_4_Al_3_Cr)_0.25_TiO_5_ phase. The diffraction spots along the zone axes [1 0 0] further confirm that the second phase has high crystallinity and pseudo‐brookite structure. Additionally, the elements distribution mapping (Figure 3h) demonstrates that the Ti element mainly distributes in the (Fe_4_Al_3_Cr)_0.25_TiO_5_ phase, suggesting that over‐saturated Ti doping prompts the generation of the whiskers.

**Figure 4:**
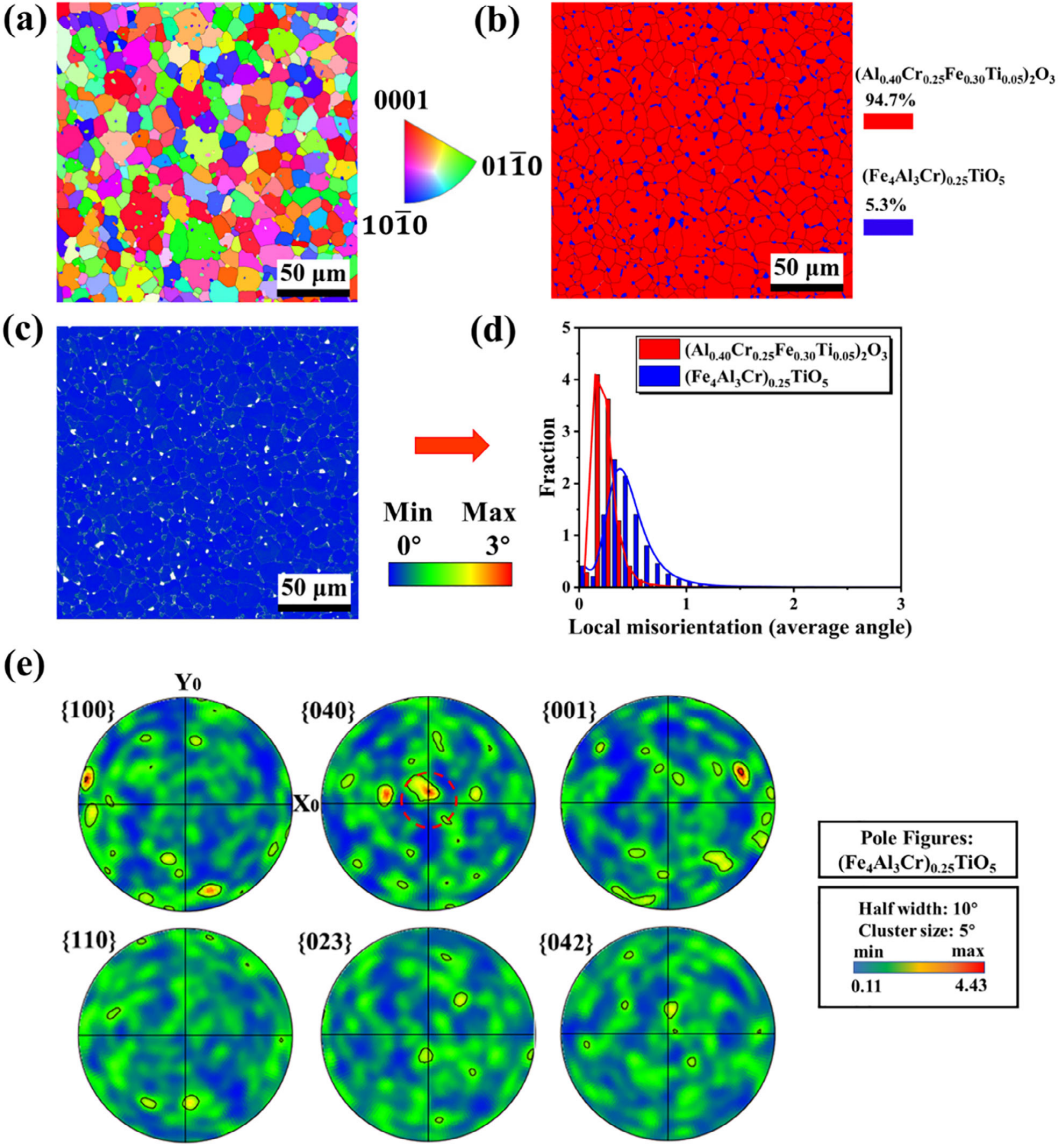
a) Inverse pole Figure (IPF‐Z_0_), b) phase distribution, c) Kernel Average Misorientation (KAM) Figure, d) local misorientation Figure, and e) pole figures of (Fe_4_Al_3_Cr)_0.25_TiO_5_.

To further investigate microstructure, EBSD of MEO‐2 prepared under oxygen partial pressure of 50.6 kPa was carried out. **Figure** **4**a shows the inverse pole figure (IPF‐Z_0_) and phase distribution of MEO‐2. Around 5.3% (Fe_4_Al_3_Cr)_0.25_TiO_5_ phase in blue color is non‐uniformly distributed at grain boundaries of (Al_0.40_Cr_0.25_Fe_0.30_Ti_0.05_)_2_O_3_ in red color, which is confirmed with the SEM results. Combined with the local misorientation figures (Figure 4c,d), the (Fe_4_Al_3_Cr)_0.25_TiO_5_ phase exhibits higher values than (Al_0.40_Cr_0.25_Fe_0.30_Ti_0.05_)_2_O_3_ phase at the misorientation angle ranging from 0° to 3°, which demonstrates the plastic deformation of (Fe_4_Al_3_Cr)_0.25_TiO_5_ phase is better than that of the (Al_0.40_Cr_0.25_Fe_0.30_Ti_0.05_)_2_O_3_ phase.^[^ ^38^ ^]^ Furthermore, Figure 4e shows the pole figure of (Fe_4_Al_3_Cr)_0.25_TiO_5_ with random distribution in {100}, {001}, {110}, {023}, {040} and {042} planes. It can be observed that the local texture strengths of {100}, {001}, and {040} planes are higher than those of {110}, {023}, and {042} planes, while the {040} plane has the maximum texture strength of 4.43, demonstrating that the (Fe_4_Al_3_Cr)_0.25_TiO_5_ phase preferentially grows along the {040} plane.^[^ ^39^ ^]^

### 3.2 The Growth Mechanism of (Fe_4_Al_3_Cr)_0.25_TiO_5_ Whisker

**Figure 5:**
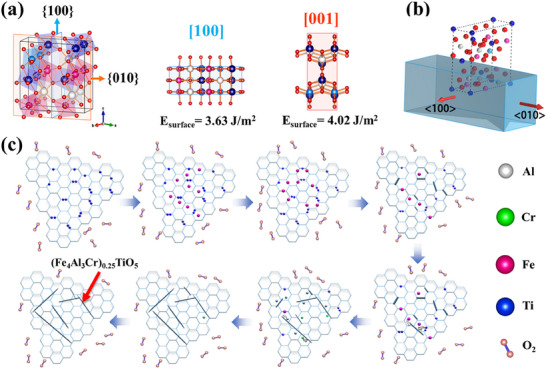
a) Pseudo‐brookite structure and the surface energy of (100) and (001) planes, b) the BFDH morphology simulation of (Fe_4_Al_3_Cr)_0.25_TiO_5_, and c) the schematic diagram of (Fe_4_Al_3_Cr)_0.25_TiO_5_ whisker growth.

The growth mechanism of whiskers must be closely related to the surface energy and plane density due to their anisotropy.^[^ ^40^ ^]^ Therefore, to better understand the growth mechanism of (Fe_4_Al_3_Cr)_0.25_TiO_5_ phase, surface energy calculation, and BFDH morphology simulation were conducted. **Figure** **5**a shows the pseudo‐brookite crystal structure, (1 0 0) and (0 0 1) planes of the (Fe_4_Al_3_Cr)_0.25_TiO_5_ phase, respectively. The surface energy of (1 0 0) and (0 0 1) planes in (Fe_4_Al_3_Cr)_0.25_TiO_5_ were 3.63 and 4.02 J m^−2^, respectively. Generally, the low surface energy planes of materials are more easily used as exposed surfaces,^[^ ^41^ ^]^ so (1 0 0) plane corresponds to the most exposed face in the morphology of whiskers. In addition, the morphology of (Fe_4_Al_3_Cr)_0.25_TiO_5_ can be calculated in the Morphology code of the Materials Studio software package using the BFDH method. The BFDH law means that the final shape of the crystal should be surrounded by the crystal plane with the highest surface density, the growth rate is inversely proportional to the interplanar spacing, and the plane with the fastest growth rate disappears in the final morphology.^[^ ^42^ ^]^ Through morphology calculation (Figure 5b), the preferred growth direction is more likely to appear in the <0 1 0> direction.

Based on the above results, the growth mechanism of (Fe_4_Al_3_Cr)_0.25_TiO_5_ phase is proposed and its schematic diagram is shown in Figure 5c. First, the excessive Ti element mainly distributes at grain boundaries and interacts with the diffusive Fe element, forming short whiskers with a pseudo‐brookite structure. Second, Al and Cr elements diffuse to grain boundaries and react with the whiskers due to their relatively low diffusion coefficient,^[^ ^43^ ^]^ facilitating whisker growth along the (0 4 0) plane. Finally, the excessive Ti element at the grain boundary is consumed, terminating whisker growth. Besides, the whiskers are related to the vacancy concentration and the valence state of the element, which can be regulated by oxygen partial pressure. When the oxygen partial pressure is low, the vacancy concentration is high, which is conducive to atomic diffusion.^[^ ^44^ ^]^ However, the Ti^3+^ and Fe^2+^ contents at low oxygen partial pressure are higher, which is not conducive to (Fe_4_Al_3_Cr)_0.25_TiO_5_ generation. When the oxygen partial pressure is too high, the Ti^4+^ and Fe^3+^ contents increase, but the vacancy concentration is significantly lower, resulting in a shorter growth length of whiskers. Therefore, reasonable regulation of oxygen partial pressure can significantly regulate the whisker growth.

### 3.3 Mechanical Properties

**Figure 6:**
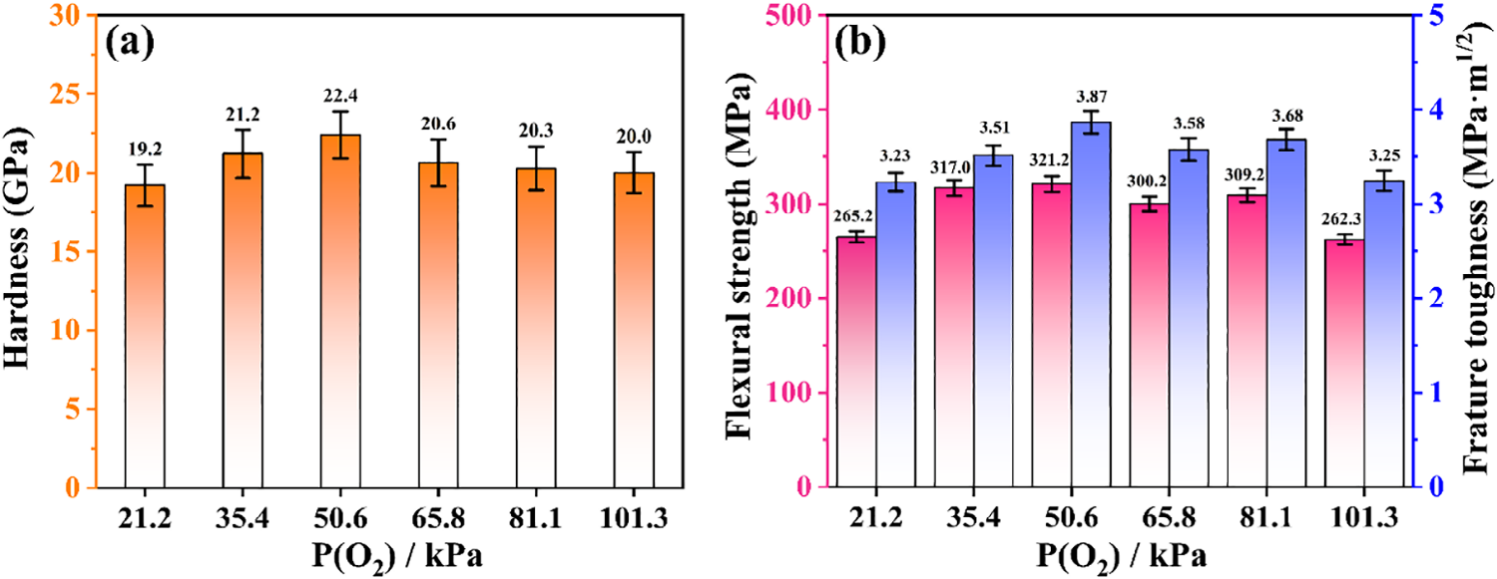
a) Hardness, b) flexural strength, and fracture toughness of MEO‐2 prepared under different oxygen partial pressures.

To evaluate the effect of oxygen partial pressure on the mechanical properties of MEO‐2, the hardness, flexural strength, and fracture toughness were measured. As shown in **Figure** **6**a, MEO‐2 prepared at different oxygen partial pressures have similar Vickers hardness values from 19.2 ± 1.2 to 22.4 ± 1.5 GPa. The maximum Vickers hardness of 22.4 ± 1.5 GPa can be obtained when the oxygen partial pressure is 50.6 kPa. Figure 6b shows the flexural strength and fracture toughness of MEO‐2. As expected, MEO‐2 prepared at oxygen partial pressure of 50.6 kPa possesses the maximum flexural strength and fracture toughness of 321 ± 8 and 3.87 ± 0.12 MPa m^1/2^, respectively. In addition, the theoretical fracture strength of MEO‐1 is only 1.37 MPa m^1/2^ (Calculation details are described in Figure S7, Supporting Information). Compared to MEO‐1, the mechanical properties of MEO‐2 controlled by oxygen partial pressure are significantly improved. The fracture toughness of MEO‐2 is much higher than the theoretical fracture toughness (1.37 MPa m^1/2^) of MEO‐1,^[^ ^45^ ^]^ which indicates the generation and regulation of in situ whiskers play a significant role in improving fracture toughness. Furthermore, the main toughening mechanism is whisker debonding and pullout based on the fracture surface of MEO‐2 (Figure S8, Supporting Information), which are attributed to the largest average length and aspect ratio of the whiskers. Therefore, the mechanical properties of corundum‐type medium‐entropy oxides can be improved significantly by in situ formed whiskers, which can be regulated facilely by the applied partial oxygen pressure.

### 3.4 Strengthening Mechanism

**Figure 7:**
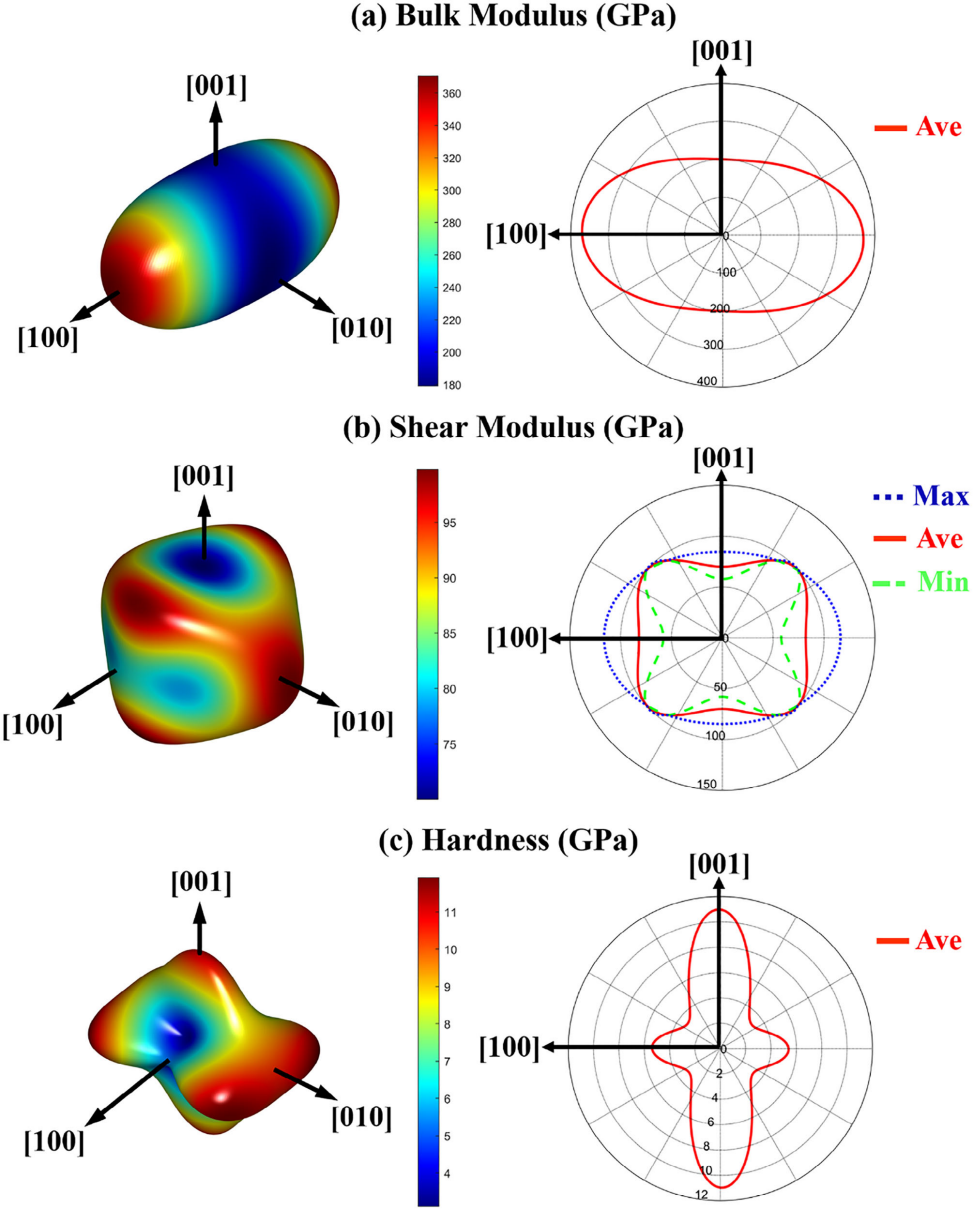
a) Anisotropic bulk modulus, b) shear modulus, and c) hardness of the (Fe_4_Al_3_Cr)_0.25_TiO_5_ phase.

To demonstrate the reinforcing mechanism of (Fe_4_Al_3_Cr)_0.25_TiO_5_ whiskers, the full sets of independent second‐order elastic constants are calculated using the geometry optimization structure of (Fe_4_Al_3_Cr)_0.25_TiO_5_.^[^ ^46^ ^]^ **Figure** **7** shows the anisotropic bulk modulus, shear modulus, and hardness of the (Fe_4_Al_3_Cr)_0.25_TiO_5_ phase. The bulk modulus of (Fe_4_Al_3_Cr)_0.25_TiO_5_ in the direction of [1 0 0], [0 1 0] and [0 0 1] are 360 GPa, 200 GPa and 170 GPa, respectively. The shear modulus of (Fe_4_Al_3_Cr)_0.25_TiO_5_ in the direction of [1 0 0], [0 1 0] and [0 0 1] are 80 GPa, 100 GPa and 70 GPa, respectively. The hardness in the [0 1 0] and [0 0 1] directions is much larger than that in the [100] direction. The above calculations show that the whisker length direction has the characteristics of small bulk modulus and large shear modulus, resulting in good deformation ability and strong shear resistance in the length direction, while the radial direction is the opposite. Therefore, the mechanism of increasing fracture toughness for MEO‐2 is due to the high deformation ability and shear resistance in the direction of whisker length. In addition, the elastic modulus is determined using Hill's bulk modulus *B_H_* and Hill's shear modulus *G_H_* by the following equations:^[^ ^47^ ^]^

(6) $$E=\frac{9B_{H}G_{H}}{3B_{H}+G_{H}}$$

To verify the accuracy of the elastic modulus calculated by DFT, a nano‐indentation test was performed (Figure S9, Supporting Information). The average elastic modulus of (Al_0.40_Cr_0.25_Fe_0.30_Ti_0.05_)_2_O_3_ and (Fe_4_Al_3_Cr)_0.25_TiO_5_ are 290.6 and 232.1 GPa, and their average nano‐hardness are 26.5 and 14.4 GPa, respectively. The results show that the elastic modulus (232.1 GPa) of the nano‐indentation test of (Fe_4_Al_3_Cr)_0.25_TiO_5_ is close to the calculated elastic modulus (235.6 GPa). The error value is 1.5% (<5%), indicating that the DFT calculations are reliable. Combined with the DFT calculations, the whisker (Fe_4_Al_3_Cr)_0.25_TiO_5_ possesses high shear resistance and good deformation in the length direction due to its anisotropic mechanical properties, thus enhancing the fracture toughness of MEO‐2.

## 4 Conclusion

In summary, single and dual‐phase corundum‐type medium‐entropy oxides were successfully synthesized by a solid‐state reaction method. The XRD and EDS results show that MEO‐1 (Al_0.41_Cr_0.26_Fe_0.31_Ti_0.02_)_2_O_3_ exhibits a single‐phase corundum structure, while MEO‐2 (Al_0.40_Cr_0.25_Fe_0.35_Ti_0.05_)_2_O_3_ involves mainly corundum phase and minor pseudo‐brookite phase (Fe_4_Al_3_Cr)_0.25_TiO_5_. By regulating oxygen partial pressures, (Fe_4_Al_3_Cr)_0.25_TiO_5_ is formed to whiskers with the maximum length and aspect ratio under oxygen partial pressure of 50.6 kPa, where the optimum Vickers hardness, flexural strength, and fracture toughness of MEO‐2 are 22.4 ± 1.5 GPa, 321 ± 8 MPa and 3.87 ± 0.12 MPa m^1/2^, respectively. The preferred growth direction of the (Fe_4_Al_3_Cr)_0.25_TiO_5_ is the [0 4 0] direction, which enhances the fracture toughness of ceramics. First‐principles calculations demonstrate that the mechanical properties of the (Fe_4_Al_3_Cr)_0.25_TiO_5_ are anisotropic and the [0 4 0] direction of the whisker has the highest shear modulus. Consequently, a novel in situ whisker‐reinforced corundum‐type medium‐entropy oxide ceramics can be designed and fabricated by controlling ion valence states, phase distribution, and growth morphology using oxygen partial pressure, which provides a new insight for the design of multi‐component and high controllability of medium/high‐entropy ceramics.

## Conflict of Interest

The authors declare no conflict of interest.

## Supporting information



Supporting Information

## Data Availability

The data that support the findings of this study are available from the corresponding author upon reasonable request.
