# Supplementary material for: Mechanisms of In Situ Growth of (Fe4Al3Cr)0.25TiO5 Whisker Driven by Oxygen Partial Pressure and Reinforced Mechanical Properties in Corundum‐Type Medium‐Entropy Oxides
Source: Adv Sci (Weinh). 2025 Jul 30;12(35):e08378. doi: 10.1002/advs.202508378 (PMC12462912; doi:10.1002/advs.202508378)
Supplement: Supplementary file 1 — Supporting Information [file ADVS-12-e08378-s001.docx]

**Supporting Information**

**Mechanisms of In-Situ Growth of (Fe_4_Al_3_Cr)_0.25_TiO_5_ Whisker driven by Oxygen Partial Pressure and Reinforced Mechanical Properties in Corundum-Type Medium-Entropy** **Oxides**

*Wenxue Wang^a, e ,1^, Kang Wang ^a,^ ^1^, Chao Ma^a,^ ^b, *^, Wei Yang^c, d^,* *Junpeng Jiang^a^, Rui Zhao^a^, Daoyang Han^a^, Hailong Wang^a^, Rui Zhang^a^*

^a^School of Materials Science and Engineering, Zhengzhou University, Zhengzhou, Henan 450001, China

^b^Zhongyuan Critical Metal Laboratory, Zhengzhou University, Zhengzhou, Henan, 450001, China

^c^State Key Laboratory of Structural Analysis, Optimization and CAE Software for Industrial Equipment, Zhengzhou University, Zhengzhou, Henan, 450002, China

^d^Zhengzhou Research Institute for Abrasives & Grinding Co., Ltd. Zhengzhou, Henan 450001, China

^e^State Key Laboratory of Inorganic Synthesis and Preparative Chemistry, College of Chemistry, Jilin University, Changchun 130012, China

^1^These two authors contributed equally to this work.

^*^Corresponding author: chaoma@zzu.edu.cn


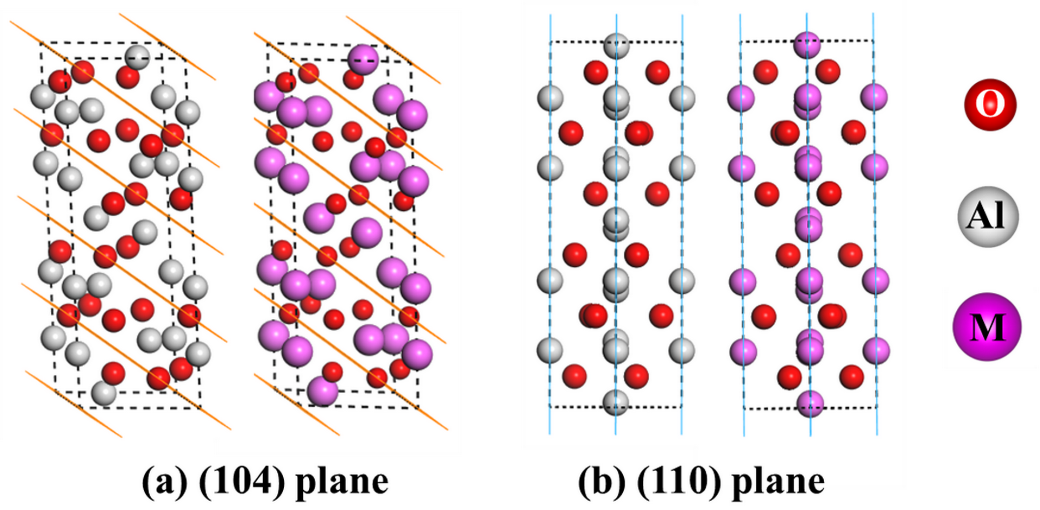


**Figure S1**. a) the (104) planes of Al_2_O_3_ and MEO-1, and b) the (110) planes of Al_2_O_3_ and MEO-1, M stands for metal atoms in MEO-1.

The intensity ratios of (104) to (110) in the experimental XRD pattern is lower than that of Al_2_O_3_ PDF card (PDF#10-0173), but higher than Cr_2_O_3_ PDF card (PDF#84-0315). To explore the reason for the change of peak intensity ratios of (104) to (110), the (104) and (110) planes of Al_2_O_3_ and MEO-1 are exhibited in Figure S1. In Al_2_O_3_ and MEO-1, the oxygen atoms are located on (104) plane, while metal atoms are located on (110) plane. Generally, the peak intensity is predominately dependent on the atomic scattering factor *f*(s) which is defined as follows^[1]^:

$\text{f}\text{(s)}\text{ }\text{=}\text{ }\text{Z}\text{ }\text{-}\text{ }\text{41.78214}\text{ }\text{×}{\text{ }\text{s}}^{\text{2}}\text{ }\text{×}\text{ }\sum_{\text{i}\text{=1}}^{\text{N}} \text{a}_{\text{i}}\text{e}^{{\text{-}\text{b}}_{\text{i}}\text{s}^{\text{2}}}$ (1)

where *Z* is the atomic number,${\text{ }\text{a}}_{\text{i}}$ and $\text{b}_{\text{i}}$ are the coefficients listed in Table , and *s* is determined as the following Equation S2^[1]^:

$\text{s}\text{ }\text{=}\text{ }\frac{\text{sinθ}}{\text{λ}}$ (2)

where *θ* and *λ* are the glancing angle and wavelength, respectively.

The *f*(s) values of selected elements on (104) and (110) planes are calculated according to Equation S1. The parameters and atomic scattering factors of selected elements are shown in Table S2. The atom scattering factor of Al is the smallest among all metal elements, while the atom scattering factor of Fe is the largest. To demonstrate the effect of substitution of other metal atoms on the peak intensity of (110) plane, the atomic scattering factor of (110) plane in MEO-1 is calculated by the following Equation S3:

$\text{M}_{\text{f(110)}} \text{=} \text{0.41}\text{Al}_{\text{f}\left( \text{110} \right)}\text{ + 0.26}\text{Cr}_{\text{f}\left( \text{110} \right)}\text{ + 0.31}\text{Fe}_{\text{f(110)}}\text{ + 0.02}\text{Ti}_{\text{f(110)}}\text{ = 14.975}$ (3)

The Cr_2_O_3_ and Fe_2_O_3_ with high atomic scattering factors have low peak intensity ratios of (104) to (110), while those with small atomics scattering factors, such as Al_2_O_3_, shows high peak intensity ratios. Thus, the relatively low (104) to (110) peak intensity ratios of the corundum-type medium-entropy oxides can be attributed to the introduction of Fe and Cr atoms with high scattering factors in the solid solutions.


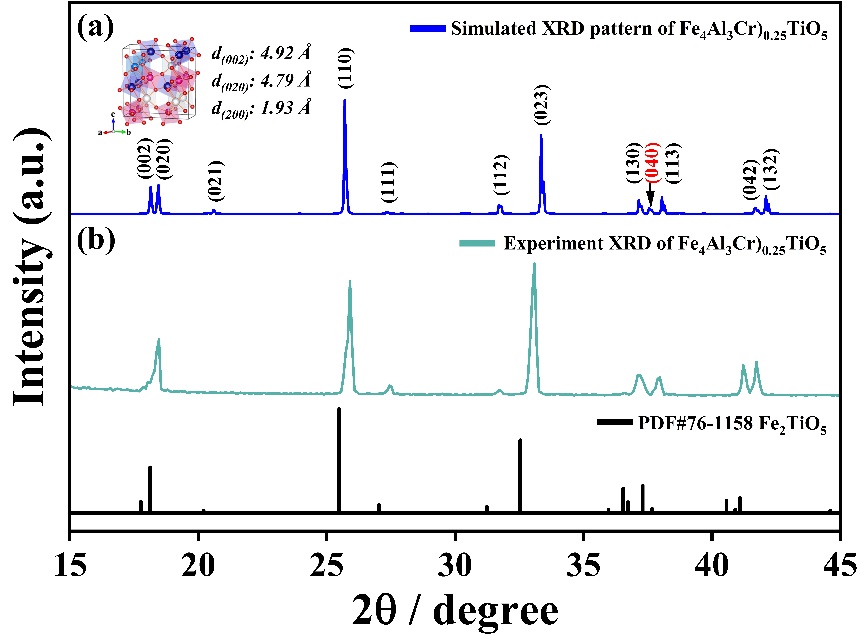


**Figure S2**. a) The simulated XRD pattern of (Fe_4_Al_3_Cr)_0.25_TiO_5_, b) experiment XRD pattern of (Fe_4_Al_3_Cr)_0.25_TiO_5_.

In Figure S2a, the XRD pattern of the (Fe_4_Al_3_Cr)_0.25_TiO_5_ supercell was simulated using VESTA software. Compared to FeTi_2_O_5_ (PDF#76-1158), the diffraction peaks of (Fe_4_Al_3_Cr)_0.25_TiO_5_ shift to higher angles due to the substitution of Fe sites by Al and Cr, which have smaller ionic radii, resulting in a reduced interplanar spacing (such as (002), (020) and (200) planes). Additionally, to confirm the reliability of the (Fe_4_Al_3_Cr)_0.25_TiO_5_ simulation phase, we synthesized it using α-Al_2_O_3_, α-Fe_2_O_3_, Cr_2_O_3_, and rutile TiO_2_ powders (99.9% purity, Rhawn Biochemical Co., Ltd., Shanghai, China) under the same conditions (1500 °C, 2 h) according to its chemical composition. Figure S2b shows the experimental XRD pattern of (Fe_4_Al_3_Cr)_0.25_TiO_5_. It can be observed that its diffraction peak shape is similar to that of Fe_2_TiO_5_, and the diffraction peaks also shift toward higher angles.


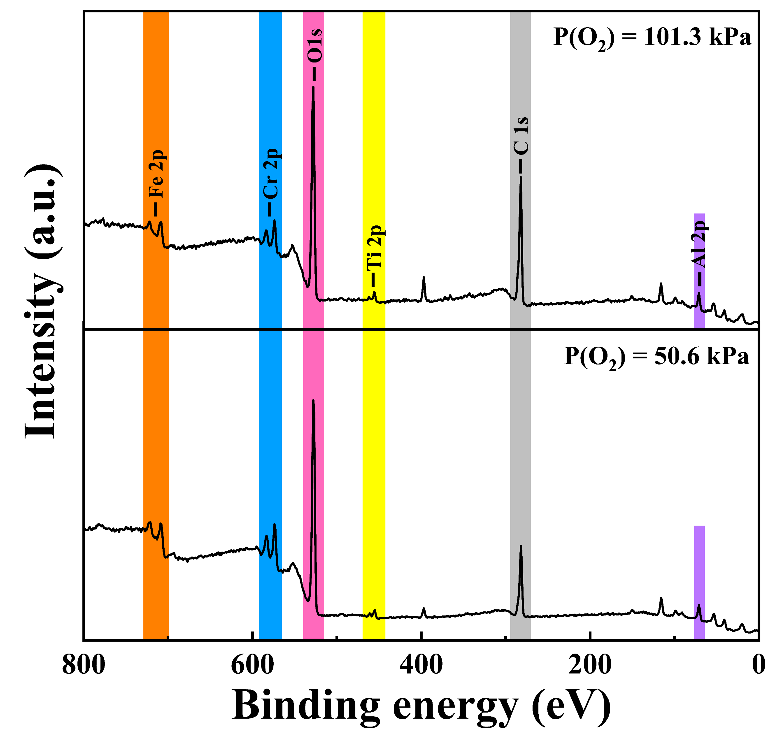


**Figure S3**. The XPS full spectrum of MEO-2 prepared under 50.6 kPa and 101.3 kPa.


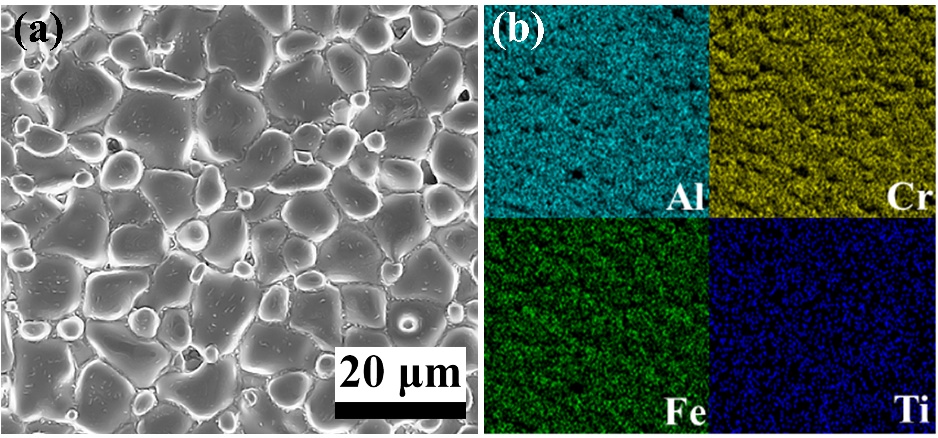


**Figure S4**. a) surface morphology and b) distribution of constituting elements of MEO-1 (Al0_.41_Cr_0.26_Fe_0.31_Ti_0.02_)_2_O_3_.

The distribution of elements in MEO-1 is uniform and without elements segregation, as shown in Figure S4. Therefore, the single-phase corundum-type medium-entropy oxide has been successfully synthesized by adding 2 at% Ti.


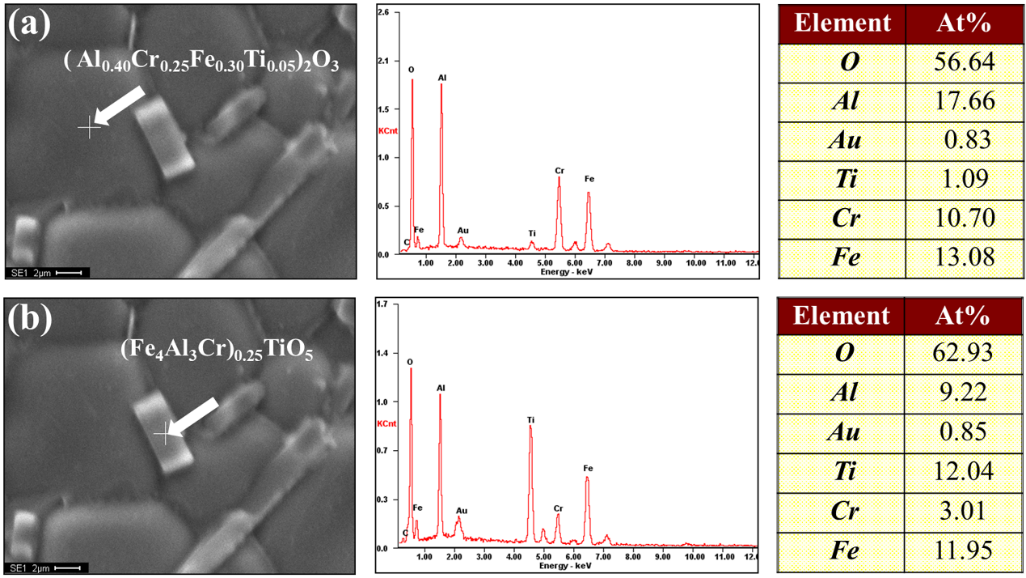


**Figure S5**. Element composition (spot scan) of a) (Al_0.40_Cr_0.25_Fe_0.30_Ti_0.05_)_2_O_3_ and b) (Fe_4_Al_3_Cr)_0.25_Ti_2_O_5_ (MEO-2 prepared at oxygen partial of 101.3 kPa).


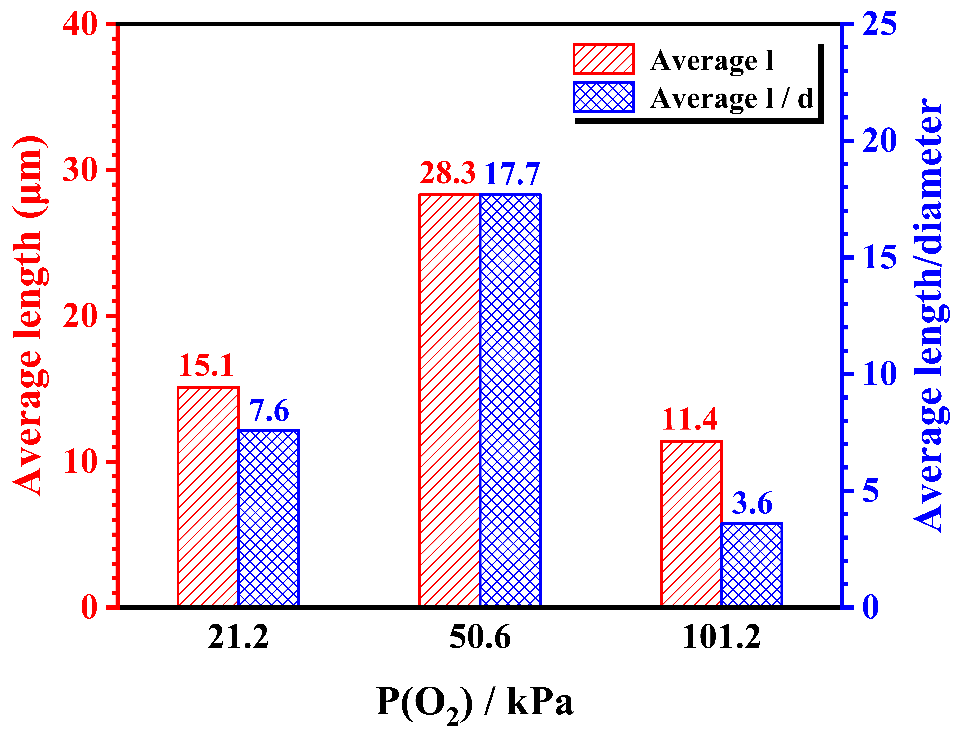


**Figure S6**. Average length and aspect ratio of the whiskers of MEO-2.


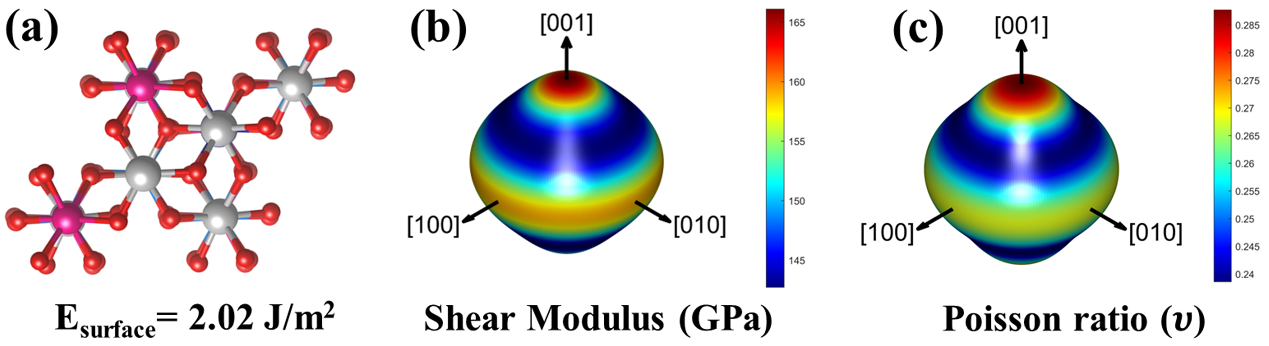


**Figure S7**. a) (001) surface energy, b) shear modulus and c) Poisson’s ratio of MEO-1.

The mechanical properties of MEO-1 are calculated by first-principles calculation. The theoretical fracture toughness of MEO-1 is calculated using the second order force constant by the following equation^[2]^:

$\text{K}_{\text{g}}=2\sqrt{\frac{\gamma_{s}G}{(1-\nu)}}$ (4)

where $\gamma_{s}$ ,$\nu$ and G are the surface energy, Poisson’s ratio, and shear modulus of the material, respectively. *K_g_* is the so-called theoretical fracture toughness, which is applicable to the materials system without defects such as dislocations and other cracks.

The selective (001) plane is low-index face and the surface energy of MEO-1 is calculated as follows^[3]^:

$\gamma= \frac{1}{2A}(E_{slab}-N{\cdot E}_{buik})$ (5)

where the A, $E_{slab}$, $E_{buik}$, and N expressed as the surface area, the total energy of (001) planes, bulk structure energy, and the A_2_O_3_ molecules amount in the slab supercell, respectively. The surface energy of (001) is 2.02 J/m^2^, as shown in Figure S7a. Figure S7b, c show the Poisson’s ratio and shear modulus of MEO-1 calculated by second order force constant, respectively. According to Equation S4, the theoretical fracture strength of MEO-1 is only 1.37 MPa·m^1/2^.


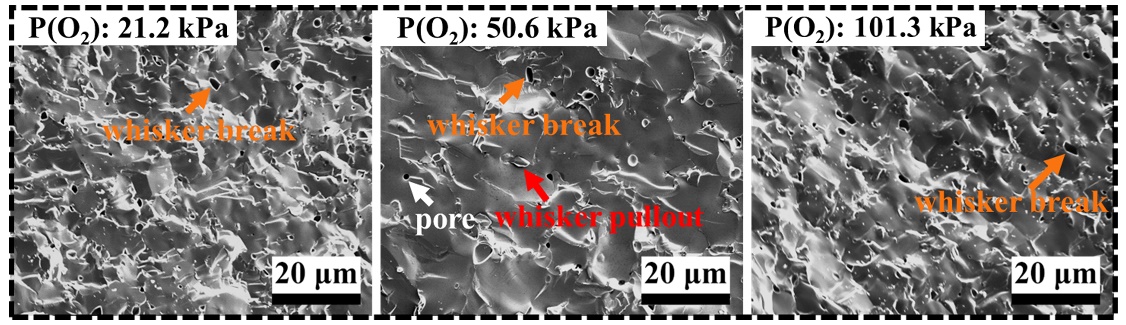


**Figure S8**. The fracture morphology of MEO-2 prepared under 21.6, 50.6 and 101.3 kPa.


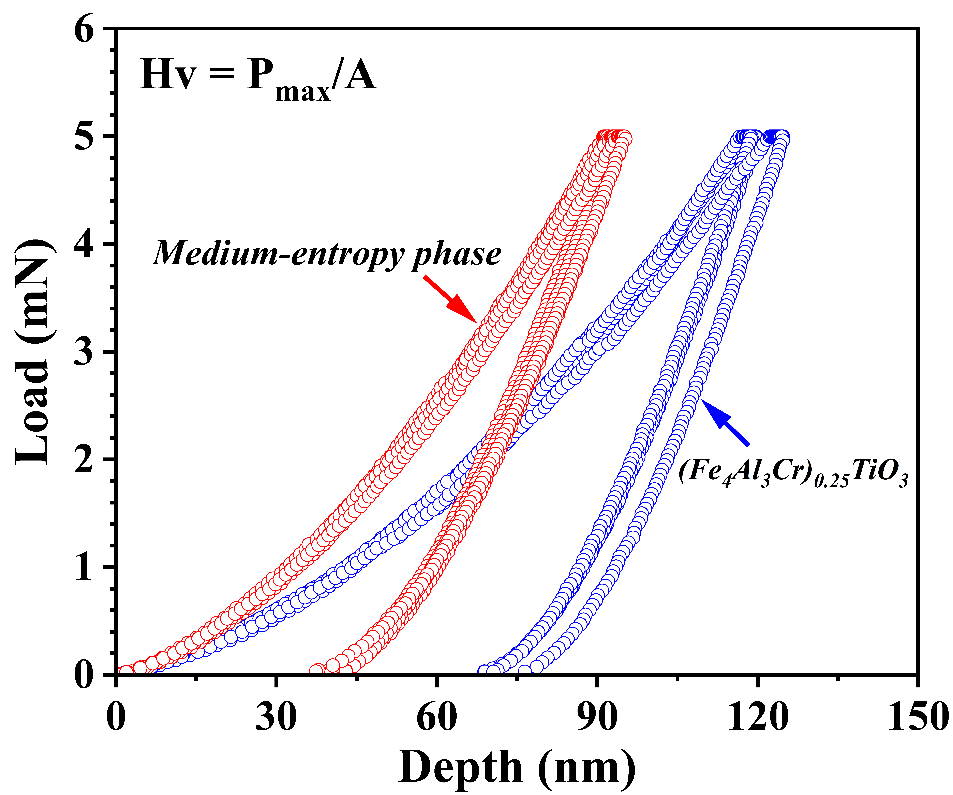


**Figure S9**. Nano-indentation load-displacement curves of MEO-2 prepared under 50.6 kPa.

Table S1 Lattice parameters and ionic radius of cations of some corundum-type oxides.

| System | Lattice parameters *a* (Å) | Lattice parameters *c* (Å) | Space group | Crystal structure | Ionic radius (Å) |
| --- | --- | --- | --- | --- | --- |
| Al_2_O_3_ | 4.810 | 13.120 | *R*$\bar{\text{3}}$*C* | Trigonal | 0.535 |
| Cr_2_O_3_ | 4.913 | 13.469 | *R*$\bar{\text{3}}$*C* | Trigonal | 0.615 |
| Fe_2_O_3_ | 5.100 | 13.910 | *R*$\bar{\text{3}}$*C* | Trigonal | 0.645(+3)/0.780(+2) |
| Ga_2_O_3_ | 5.060 | 13.620 | *R*$\bar{\text{3}}$*C* | Trigonal | 0.620 |
| Sc_2_O_3_ | 5.370 | 14.390 | *R*$\bar{\text{3}}$*C* | Trigonal | 0.745 |
| Ti_2_O_3_ | 5.110 | 13.980 | *R*$\bar{\text{3}}$*C* | Trigonal | 0.670(+3)/0.605(+4) |
| TiO_2_ | 4.650 | 2.970 | *P4₂/mnm* | Rutile | 0.605(+4) |
| In_2_O_3_ | 5.590 | 14.750 | *R*$\bar{\text{3}}$*C* | Trigonal | 0.760 |
| V_2_O_3_ | 5.130 | 14.130 | *R*$\bar{\text{3}}$*C* | Trigonal | 0.780 |
| Mn_2_O_3_ | 5.160 | 14.010 | *R*$\bar{\text{3}}$*C* | Trigonal | 0.645 |
| Rh_2_O_3_ | 5.210 | 14.090 | *R*$\bar{\text{3}}$*C* | Trigonal | 0.820 |

Table S2 Atomic scattering factor *f* and parameters for selected elements [1].

| Element | Z | a_1_ | b_1_ | a_2_ | b_2_ | a_3_ | b_3_ | a_4_ | b_4_ | S_104_ | f_104_ | S_110_ | f_110_ |
| --- | --- | --- | --- | --- | --- | --- | --- | --- | --- | --- | --- | --- | --- |
| O | 8 | 0.455 | 23.780 | 0.917 | 7.6220 | 0.472 | 2.144 | 0.138 | 0.296 | 0.195 | 5.709 | 0.210 | 5.456 |
| Al | 13 | 2.276 | 72.322 | 2.428 | 19.733 | 0.858 | 3.080 | 0.317 | 0.408 | 0.195 | 9.238 | 0.210 | 8.999 |
| Cr | 24 | 2.307 | 78.405 | 2.334 | 15.785 | 1.823 | 3.157 | 0.490 | 0.364 | 0.189 | 18.656 | 0.203 | 18.159 |
| Fe | 26 | 2.544 | 64.424 | 2.343 | 14.880 | 1.759 | 2.854 | 0.506 | 0.350 | 0.186 | 20.533 | 0.198 | 20.131 |
| Ti | 22 | 3.565 | 81.982 | 2.818 | 19.049 | 1.893 | 3.590 | 0.483 | 0.386 | 0.182 | 16.612 | 0.195 | 16.199 |

**References**

[1] G. S. Rohrer, *Structure and bonding in crystalline materials*, Cambridge University Press. , **2001**.

[2] Z. Ding, S. Zhou, Y. Zhao, *Phys. Rev. B* **2004**, *70* (18), 184117, https://doi.org/10.1103/PhysRevB.70.184117.

[3] B. Li, Y. Duan, M. Li, M. Peng, S. Zheng, *Mater. Chem. Phys.* **2024**, *313*, 128771, https://doi.org/10.1016/j.matchemphys.2023.128771.
